# Investigation of Antidiabetic Effect of *Pistacia atlantica* Leaves by Activity-Guided Fractionation and Phytochemical Content Analysis by LC-QTOF-MS

**DOI:** 10.3389/fphar.2022.826261

**Published:** 2022-02-25

**Authors:** Sultan Pekacar, Didem Deliorman Orhan

**Affiliations:** Department of Pharmacognosy, Faculty of Pharmacy, Gazi University, Ankara, Turkey

**Keywords:** α-amylase, antidiabetic, antioxidant, α-glucosidase, LC-QTOF-MS, pancreatic cholesterol esterase, Pistacia atlantica, RP-HPLC

## Abstract

In this study, the antidiabetic, antiobesity, antioxidant, and antihyperlipidemic effects potential of *Pistacia atlantica* Desf. leaves were evaluated by *in vitro* methods. The effects of the leaves of the plant on pancreatic lipase, pancreatic cholesterol esterase, and PTP1B enzymes were investigated for the first time and it was observed that leaf methanol extract (IC_50_: 123.67 ± 0.40 μg/ml) and *n*-hexane sub-extract (IC_50_: 61.03 ± 0.11 μg/ml) had much stronger effects on pancreatic cholesterol esterase enzyme than simvastatin (IC_50_: 142.30 ± 5.67 μg/ml). The methanolic extract of *P. atlantica* leaves exerted strong inhibitory effect on the enzymes (*α*-amylase and *α*-glucosidase) effective on carbohydrate digestion. It was thought that the methanol extract could provide significant benefits against oxidative stress in diabetes mellitus since it showed antioxidant activities (DPPH radical scavenging activity and reducing power) as strong as reference compounds (ascorbic acid and quercetin). Qualitative and quantitative analyzes of rutin (0.328 ± 0.000 g/100 g dry extract), methyl gallate (5.245 ± 0.014 g/100 g dry extract), quercetin-3-*O-*glucoside (0.231 ± 0.000 g/100 g dry extract), and gallic acid (0.528 ± 0.127 g/100 g dry extract) in methanol extract were performed by RP-HPLC. The phytochemical content of the active sub-fraction obtained from the leaf methanol extract by activity-guided fractionation and column chromatography studies was characterized by LC-QTOF-MS. The presence of trigalloylglucose, digalloylglucose, and methyl gallate in the G6 coded sub-fraction obtained by chromatographic techniques from the ethyl acetate sub-extract, which has the highest inhibitory effect on *α*-amylase and *α*-glucosidase enzymes, was determined by LC-QTOF-MS. In addition to the G5 coded subfraction, a strong *α*-glucosidase enzyme inhibitory activity was also observed in the G6 coded sub-fraction, and methyl gallate, methyl digallate, 2″-*O*-galloyl-quercetin-3-*O*-hexoside, and myricetin-3-*O*-hexoside were identified in this sub-fraction. This study displayed that the methanol extract of *P. atlantica* leaves could be a potential source for bioactive compounds with antidiabetic effects by showing inhibitory effects on enzymes involved in carbohydrate digestion.

## Introduction

Diabetes is one of the important health problems affecting people worldwide. It is reported that the World Health Organization (WHO) predicts that approximately 300 million people will have diabetes by 2025. Obesity is among the most important and major risk factors for diabetes. It is reported that approximately 60–90% of patients with type 2 diabetes are obese. Several clinical studies have reported an association between obesity and insulin resistance in adults. There are also reports suggesting that weight loss is associated with a decrease in insulin concentration and an increase in insulin sensitivity in children, adolescents, and adults (Siddiqui, 2018). Therefore, in the fight against diabetes mellitus, which is a common metabolic disorder; there is a need for new drugs of natural or synthetic origin that can lower blood sugar, reduce oxidative stress caused by diabetes, and play a role in weight control. Plants used against diabetes in traditional folk medicine in various geographical regions are the subject of inspiring studies for many researchers to discover new drug molecules.

*Pistacia* L. is a genus belonging to the Anacardiaceae family, comprising about 70 genera and more than 600 species (Ahmed et al., 2021). *Pistacia atlantica* Desf. it is one of the important species of this genus, which is widely grown in the Mediterranean and Middle East regions. *P. atlantica* is a light fragrant tree 2–5 m tall, with grayish brown branches, 9–11 leaflets, and a yellowish-green oleoresin can be obtained from its trunk (Mahjoub et al., 2018). As a result of phytochemical studies on *P. atlantica*, it has been reported that it contains many compounds including volatile compounds, flavonoids, phenolic compounds, fatty acids, tocopherols, and phytosterols. Many scientific studies have shown that crude extracts of *P. atlantica* or compounds isolated from the extracts have a wide variety of pharmacological activities such as antimicrobial, antifungal, anti-inflammatory, analgesic, antinociceptive, anticancer, cytotoxic, anticholinesterase, antidiabetic, hepatoprotective, urease inhibitory, antihypertension, wound healing, nipple fissure healing, antileishmanial and antiplasmodial activities (Ahmed et al., 2021). In the literature, it has been reported that the resin, leaf, fruit, and aerial parts of *Pistacia* species are traditionally used in the treatment of digestive system, kidney, heart, and respiratory tract diseases and as wound healing. In addition, there is information in the ethnobotanical records of Jordan that *P. atlantica* leaves are traditionally used in diabetes mellitus (Bozorgi et al., 2013).

Since the *in vitro* antidiabetic activity results of *P. atlantica* crude extract, which were previously performed in our laboratory, were promising, this study aimed to examine the antidiabetic and antiobesity effects in more detail. For this purpose, activity-guided fractionation was carried on the leaves of *P. atlantica* and antidiabetic and antiobesity potentials of both the crude methanol extract and the sub-extracts with various polarities were evaluated. The antidiabetic (*α*-amylase and *α*-glucosidase inhibitory activities) and antiobesity (pancreatic lipase inhibitory activity) potentials of the methanolic extract of *P. atlantica* leaves and the sub-extracts obtained from this crude extract by using liquid-liquid fractionation method were investigated *in vitro*. In addition, the inhibitory activities of the crude extract and all sub-extracts on pancreatic cholesterol esterase and Protein tyrosine phosphatase 1B (PTP1B) enzymes were investigated by *in vitro* methods. While the antioxidant activities of the crude extract were determined by the total antioxidant capacity test, metal chelation, reducing power test, and 2,2-diphenyl-1-picrylhydrazil (DPPH) scavenging activity tests, the total phenol and total flavonoid contents of the crude extract were evaluated using the UV spectroscopic method. Fractions obtained from the most active ethyl acetate sub-extract using different column chromatography techniques (polyamide and RP-18 columns) were tested for all activities. The phytochemical contents of the subfractions obtained by column chromatography methods were determined using Qualitative tandem liquid chromatography quadrupole time of flight mass spectrometry (LC-QTOF-MS) method. the ethyl acetate sub-extract and the crude extract were standardized by reversed-phase high-performance liquid chromatography (RP-HPLC) with diode array detection on some phenolic compounds as markers.

## Materials and Methods

### General Experimental Procedures

Mass spectrometry analysis was performed with Agilent G6550A LC-QTOF-MS (Agilent Technologies, Inc., CA, United States). RP-HPLC analysis was performed with Agilent 1260 Infinity equipped with a diode array detector (DAD). VersaMax (VersaMax Molecular Devices, Sunnyvale, CA, United States) ELISA Microplate Reader and Spectramax i3x (Spectramax i3x Molecular Devices, San Jose, CA, United States) were used for the measurement of absorbance to determine the enzyme inhibitions. Polyamide (Merck KGaA, Darmstadt, Germany for column chromatography, 6), LiChroprep^®^ RP-18 (Merck KGaA, Darmstadt, Germany, 25–40 µm) were used for column chromatography. Silica gel 60 (Merck, Darmstadt, Germany, F_254_ 0.2 mm) plates were used for thin layer chromatography studies (TLC). After this process the plates were dried and spots were detected on TLC under UV_254_ and UV_366_ or by heating after spraying the plates with sulfuric acid (5%). Solvents, chemicals used in HPLC and LC-QTOF-MS analysis, and all enzymes and substrates were purchased from Sigma-Aldrich (St. Louis, MO, United States) and Carlo Erba (Milan, Italy) companies. In addition, Acarbose, orlistat, and simvastatin were purchased from Bayer Group (Istanbul-Turkey), Roche (Basel, Switzerland), and Nobel (Düzce, Turkey), respectively.

### Plant Materials

*P. atlantica* leaves were collected in July and September 2019 from Menemen, İzmir-Turkey. The plant was identified by Erdinç Oğur (Aegean Agricultural Research Institute, İzmir-Turkey) and voucher specimens were stored in the Herbarium of Faculty of Pharmacy- Gazi University (GUEF 3620). Plant materials were dried in the shade at room temperature and were ground with a mechanical grinder until they became homogeneous powder.

### Extraction and Column Chromatography Studies

Dried and ground *P. atlantica* leaves (300 g) were extracted with 100% methanol (2.5 L) for 24 h using a mechanical mixer (RW20, IKA Janke Kunkel Labortechnik, IKA^®^-Werke GmbH & Co. KG, Deutschland, Germany). This process was repeated five times and the extract, filtered through a filter paper each time, was concentrated to dryness in a rotary evaporator at a temperature not exceeding 45°C (Crude extract yield (MeOH extract): 47.4% w/w dry plant). The crude extract (114 g) was suspended in 90% methanol and then fractionated with solvents of increasing polarity with *n*-hexane (HE, 500 ml × 20), chloroform (CHCl_3_, 400 ml × 10), ethyl acetate (EtOAc, 400 ml × 40), and *n*-butanol/saturated with water (*n*-BuOH, 200 ml × 24). All sub-extracts were evaporated under reduced pressure at a temperature not exceeding 45°C. After all this fractionation process, the remaining aqueous (R-H_2_O) phase was dried in a lyophilizer at −80°C. A total of five sub-extracts (*n*-hexane 10.96 g, CHCl_3_ 1.00 g, EtOAc 35.99 g, *n*-BuOH 10.33 g and R-H_2_0 11.15 g) were obtained for further studies. Based on the results of enzyme inhibitory activities, it was decided to perform an activity-guided isolation study with the EtOAc sub-extract. EtOAc sub-extract (10 g) was fractionated by polyamide column chromatography using water, water-methanol mixtures prepared in different ratios, and methanol as the mobile phase. This column system was started with 100% water and finished with 100% methanol. After passing 250 ml of 100% water through the column, 250 ml of 90% water, 250 ml of 80% water, 350 ml of 75% water, 850 ml of 50% water, 1 L of 25% water, and finally 1 L of 100% methanol, in total 3950 ml. solvent was passed. The fractions obtained were evaluated continuously at UV_254_ and UV_366_ nm by using thin-layer chromatography (Mobile phase: CHCl_3_-MeOH-H_2_O, 61:32:7) and fractions with similar chromatographic profile were combined. According to the enzyme activity results, G-coded fraction (1 g) obtained from the polyamide column was applied to RP-18 column chromatography using water, water-methanol mixtures prepared in different ratios, and methanol as the mobile phase. This column system was also started with 100% water and finished with 100% methanol. After passing 350 ml of 100% water through the column, 400 ml 95% water, 400 ml 90% water, 400 ml 85% water, 350 ml 80% water, 300 ml 75% water, 300 ml 70% water, 300 ml 65% water, 300 ml 60% water, 300 ml 55% water, 300 ml 50% water, 300 ml 45% water, 200 ml 40% water, 100 ml 35% water, 200 ml 30% water, 200 ml 20% water, 200 A total of 5,300 ml of solvent was passed by passing ml of 10% water and finally 400 ml of 100% methanol. A total of six sub-fractions were obtained from both fractions. All sub-fractions obtained were evaluated for enzyme activities.

### Antioxidant Activity Assays

#### Total Antioxidant Capacity

First, after adding molybdate reagent solution to MeOH extract, the tubes were vortexed. After this procedure, the tubes were incubated at 90°C for 90 min. The absorbances of the samples were measured with an ELISA microtiter plate reader at 695 nm. Results are expressed as mg ascorbic acid equivalent (AAE)/g extract. Calibration curve equation was; y = 1.5828x + 0.1354 and the coefficient of determination was *r* ^2^ = 0.9995 (Orhan et al., 2017).

### Metal Chelating Capacity

In this method, the chelating effect of the extracts on the Fe^+2^ ion is investigated. The extract was incubated with FeCl_2_ solution (2 mM). The reaction was started by adding 0.02 ml of ferrozine solution (5 mM) and the total volume was made up to 130 µl with ethanol. The absorbances of the extracts and reference compound were measured after 10 min at 562 nm with an ELISA microtiter plate reader. The percent inhibition of formation of the ferrozine-Fe^+2^ complex was calculated using this formula. Metal chelating capacity (%) = [(A_Control_–A_Sample_)/A_Control_] × 100. Ethylenediaminetetraacetic acid (EDTA) was used as the reference compound in this experiment (Dinis et al., 1994).

### Reducing Power Assay

Samples (quercetin and MeOH extract: 2, 1, 0.5 and 0.25 mg/ml) were mixed with phosphate buffer (0.1 mol/L, pH 7.2) and K_3_Fe(CN)_6_ solutions. After the mixture was incubated at 37°C for 60 min, trichloroacetic acid solution was added to the mixture. Following centrifugation, the supernatant was mixed with the same amount of distilled water and FeCl_3_ solution. Then, FeCl_3_ solution was added to this mixture. After this process, the first measurement was made at 700 nm and then FeCl_3_ solution was added and the second measurement was made in the same wavelength ELISA microtiter plate reader and the reduction potential was calculated based on the difference. Quercetin was used as the reference compound in this experiment (Orhan et al., 2017).

### DPPH Radical Scavenging Activity

The DPPH radical scavenging activity of the MeOH extract was determined by the method described in a 96-well plate. The extract was mixed with DPPH solution and incubated for 30 min in a dark environment. The absorbance of the extracts and of the ascorbic acid used as the reference compound was then measured at 520 nm with an ELISA microtiter plate reader. DPPH radical scavenging activity inhibition (%) = [(A_Control_ – A_Sample_)/A_Control_] × 100 (Jung et al., 2011).

### Enzyme Assays

#### *α*-Amylase Inhibitory Activity

In this method, in which the potential of antidiabetic effect was evaluated, the extracts, sub-extracts, fractions, and sub-fractions were dissolved in 80% methanol. All samples and the reference compound acarbose (15 μl) were incubated with *α*-amylase from porcine pancreas type IA (0.25 U/ml pH 6.9 in 20 mM phosphate buffer) at room temperature. Potato starch (2.5%, w/v) in phosphate buffer solution (pH 6.9) was used as the substrate solution and was added to the previous mixture after 5 min at room temperature. After 15 min, 3, 5-dinitrosalicylic acid solution (color reagent) was added to the mixture and the reaction was carried out at 80°C for 40 min. Cold water is then added to all wells and the total volume is made up to 150 µl. The change in absorbance due to maltose formation was read at 540 nm by an ELISA microtiter plate reader and calculations were calculated according to the equation: A_Sample or Control_ = A_Sample_ − A_Blank_ Depending on the amount of maltose formed, a calibration curve of maltose was created and the percent inhibition was determined. Inhibition (%) = (1−(average of maltose formed in test samples/average of maltose formed in the control)) × 100. This enzyme assay was performed using a minor modification of the method reported in the literature (Ali et al., 2006).

### *α*-Glucosidase Inhibitory Activity

Primarily, *α*-glucosidase from *Bacillus stearothermophilus* type IV (was dissolved in phosphate buffer solution (0.5 M, pH 6.5). The extract, sub-extracts, fractions, and sub-fractions were dissolved in 80% methanol. All samples and reference compound (Acarbose) were incubated with enzyme in a 96-well microplate at 37°C for 15 min. After this, 20 mM 4-nitrophenyl-*α*-*D*-glucopyranoside (PNG), was added as substrate and the reaction was carried out at 37°C for 35 min. In reaction medium with a total volume of 100 μl, the increase in absorption at 405 nm due to hydrolysis of PNG by *α*-glucosidase was measured with an ELISA microtiter plate reader. The percent inhibition was calculated according to the following equation: Inhibition (%) = (1−(Z−z/A−a)) × 100 what is stated here can be expressed as: a is the negative control without inhibitor, A is the activity without inhibitor, z is the negative control with inhibitor, and Z is the activity with inhibitor. This enzyme assay was performed using a minor modification of the method reported in the literature (Lam et al., 2008).

### Pancreatic Lipase Inhibitory Activity

The slightly modified method of Lee et al. (2012) was used to evaluate the anti-obesity effect potential of the extract, sub-extracts, and fractions. Extract, sub-extracts, fractions and reference compound Orlistat (20 μl) were incubated with porcine pancreatic lipase type II solutions (in 10 mM MOPS (4-morpholinepropanesulfonic acid) and 1 mM EDTA buffer, 1 mg/ml) in Tris buffer (100 mM Tris-HCl and 5 mM CaCl_2_, pH 7.4) in a 96-well microtiter plate for 15 min at 37°C. Then, 4-nitrophenyl butyrate (10 mM in acetonitrile) used as substrate was placed in the wells and the reaction was carried out at 37°C for 30 min. The total volume of reaction medium in this experiment was 200 µl. The absorbance of the *p*-nitrophenol formed as a result of the experiment was evaluated at a wavelength of 405 nm. The percent inhibition was calculated according to the following equation: Inhibition (%) = (1−(Z−z/A−a)) × 100 what is stated here can be expressed as: a is the negative control without inhibitor, A is the activity without inhibitor, z is the negative control with inhibitor, and Z is the activity with inhibitor. This enzyme assay was performed using a minor modification of the method reported in the literature (Lee et al., 2012).

### Pancreatic Cholesterol Esterase Inhibitory Activity

n this method, in which the inhibition of the extracts or fractions on the pancreatic cholesterol esterase enzyme was evaluated, the extract, the fraction and the reference compound simvastatin were dissolved in 80% methanol. The extracts, fractions and reference compound (20 µl) were added to phosphate buffer. To this mixture, taurocholic acid (12 mM) and 4-nitrophenyl butyrate (5 mM in dimethylformamide) were added and after 5 min of incubation at room temperature, porcine pancreatic cholesterol esterase in phosphate buffer (100 mM pH 7.0) was added. After these procedures, a 15-min kinetic measurement was made at 405 nm. The percent inhibition was calculated as follows: Inhibition (%) = [(A_Control_ – A_Sample_)/A_Control_] × 100. This enzyme assay was performed using a minor modification of the method reported in the literature (Ngamukote et al., 2011).

### Protein Tyrosine Phosphatase 1B (PTP1B) Inhibitory Activity

The slightly modified method of Song et al. (2017) was used to evaluate the antidiabetic effect potential of the extract and sub-extracts. In this method, inhibition of PTP1B, a negative regulator of leptin and insulin signaling pathways, was evaluated. Extracts, sub-extracts, fractions, and ursolic acid used as reference compound were added to Tris-HCl buffer (pH 7.5). After 40 µl of added substrate, this mixture was allowed to incubate for 5 min. Human recombinant PTP1B was then added to the mixture in the 96-well plate (Biovision, Milpitas, CA, United States). In the ELISA microtiter plate reader set to 37°C, kinetic measurement was made at 405 nm and the percent inhibition was calculated with the following formula. Inhibition (%) = [(A_Control_ – A_Sample_)/A_Control_] × 100.

### Determination of Total Phenolic Content

MeOH extract was mixed with Folin-Ciocalteu reagent (10% w/v) and was incubated for 5 min at room temperature. Afterwards, sodium carbonate solution was added to the mixture and vortexed. The absorbance of the mixture was measured at 735 nm with an ELISA microtiter plate reader after 30 min of incubation at room temperature in a dark place. Total phenolic content was expressed as mg gallic acid equivalent (GAE)/g extract. Calibration curve equation was; y (Abs.) = 6.2614 × (Conc.) + 0.0346 and the coefficient of determination was *r* ^2^ = 0.9993 (Zongo et al., 2010)

### Determination of Total Flavonoid Content

The dried crude extract was dissolved in methanol solution (80% w/v). Ethanol, sodium acetate and aluminum chloride solutions were added to the samples and this mixture was diluted with distilled water to a total of 1 ml. After 30 min of incubation at room temperature, the absorbance of this mixture was measured at 415 nm with the ELISA microtiter plate reader. Results were expressed in mg of quercetin equivalents (QE)/g extracts. Calibration curve equation was; y (Abs) = 2.216 × (Conc.) – 0.0042 and the coefficient of determination was *r* ^2^ = 1 (Kosalec et al., 2004).

### Analysis of MeOH Extract and EtOAc Sub-extract by RP-HPLC

Both MeOH extract and the most active EtOAc sub-extract were analyzed in terms of phenolic substances by RP-HPLC method. The following standard compound mixtures were used for qualitative and quantitative analysis of phenolic acids and flavonoids. The phenolic acid mixture used for analysis includes: Gallic acid, protocatechuic acid, chlorogenic acid, vanillic acid, syringic acid, methyl gallate, *p*-coumaric acid, ferulic acid, sinapic acid, *trans*-cinnamic acid, and rosmarinic acid. The flavonoid mixture used for analysis includes: Rutin, naringenin, hesperidin, quercetin-3-*O*-glucoside, apigenin-7-*O*-glucoside, myrcetin, quercetin, luteolin, apigenin, epicatechin, and catechin. The most active EtOAc sub-extract was standardized for methyl gallate, which was found to be the major compound. Methyl gallate was previously isolated from the leaves of *Rhus coriaria* L. (Gök et al., 2020). For the analysis, HP Agilent 1260 series LC System and Agilent TC-C18 170Å (4.6 mm × 150 mm × 5 µm) column were used. The temperature of the column was 25°C throughout the analysis. The gradient system was started from the mobile phase contained 5% solvent A (acetonitrile: water: formic acid, 80:20:0.1) and 95% solvent B (water: formic acid, 100:0.1) to 100% solvent A for the 53 min. The gradient system for EtOAc sub-extract, crude extract and methyl gallate analysis is as follows: 0–10 min, 5–15% A, 0.8 ml/min flow; 10–17.01 min, 15% A, 0.6 ml/min flow; 22.01–32 min, 20–30% A, 0.8 ml/min flow; 50–53 min, 100% A, 1 ml/min flow. Detection was carried out with a DAD detector at 260, 280, 320 and 350 nm wavelengths.

### Characterization of Sub-fractions by LC-QTOF-MS Analysis

LC-QTOF-MS system was used to analyze the phytochemical contents of the sub-fractions obtained by RP-18 column chromatography. Analysis was done with Agilent 1260 series HPLC system and Agilent 6550 iFunnel High Resolution Mass Spectrometer device connected to this system. MS system was used in the double spray Agilent Jet Stream Electrospray ionization technique. Analyze was made in negative ion mode. MS operating mode is 2 GHz Extended Dynamic Range. Agilent TC-C18 170Å (4.6 mm × 150 mm × 5 µm) column was used for chromatographic separation. Agilent Mass Hunter Software B06.00 was used in the analysis. LC-QTOF-MS analysis parameters of G coded sub-fractions [Column: TC-C18 170Å (4.6 mm × 150 mm × 5 μm; Column oven: 30°C; Injection amount: 10 μl; Analysis time: 82 min; Mobile phase A: 0.1% acetic acid (v/v); Mobile phase B: Acetonitrile; Flow: 0.65 ml/min; Flow program; 0 min-5% B, 4 min-5% B, 12 min-10% B, 15 min-10% B, 28 min-20% B, 48 min-40% B, 60 min-60% B, 65 min-70% B; 66 min-90% B; 72 min-90% B; 72.10 min-5% B; UV: 260 nm (20 Hz); Ionization mode: Negative; Drying gas temperature: 200°C; Drying gas flow, nitrogen: 14 L/min; Nebulizer: 40 psi; Sheath gas temperature: 375°C; Sheat gas flow, nitrogen: 11 L/min; Capillary voltage: 1100 V; Nozzle voltage: 2000 V; Mass reading range: 30–1700 amu; Reference ions: 980.0147, 1033.9881. Data evaluation and identification were done using Agilent Masshunter Software and Metlin Metabolite database and *m/z* values, fragment ions and retention time (if standards are available) (Kılıc et al., 2019).

### Statistical Analysis

All analyzes were performed in triplicate and the results were averaged. All values are expressed as mean ± standard deviation (S.D.), linear regression analyzes and calculations were made using Microsoft Excel and GraphPad Instat software. A difference in the values of *p* < 0.05 was considered to be statistically significant (*p < 0.05, **p < 0.01, ***p < 0.001). The correlation coefficient was calculated using Microsoft Excel 2013.

## Results

### Antioxidant Activity

#### Total Antioxidant Capacity Assay

The total antioxidant capacity of the MeOH extract was evaluated, and the results were expressed as mg ascorbic acid equivalent (AAE)/g extract. The total antioxidant capacity was measured 25.86 ± 4.06 mg AAE/g extract.

### Metal Chelating Capacity

The metal chelating activity of the methanolic leaf extract of *P. atlantica* was evaluated at four different concentrations and the results were compared with EDTA used as the reference compound. EDTA showed an extremely potent effect (>100.00%) at concentrations of 1 and 2 mg/ml. It was determined that the metal chelating capacity % of the MeOH extract varied between 13.48 ± 1.16 and 48.67 ± 1.22 dose-dependently. Although the metal chelating activity of the extract increased in a dose-dependent manner, the potency was weaker than EDTA (Table 1).

**TABLE 1:** Results of DPPH radical scavenging activity, metal chelating capacity and reducing power absorbance of *P. atlantica* MeOH extract.

Samples	Concentration (mg/ml)	DPPH radical scavenging Inhibition % ± S.D.	Metal chelating capacity % ± S.D.	Reducing power absorbance ± S.D.
MeOH extract	0.25	91.31 ± 0.58^***^	13.48 ± 1.16^***^	3.061 ± 0.010^***^
	0.5	90.60 ± 0.81^***^	14.64 ± 1.66^***^	3.930 ± 0.000^***^
	1	91.18 ± 0.88^***^	25.32 ± 2.73^***^	3.927 ± 0.000^***^
	2	90.43 ± 0.13^***^	48.67 ± 1.22^***^	3.923 ± 0.000^***^
References	AA/EDTA/QE 0.25	91.61 ± 0.15^a^ ^***^	99.69 ± 0.18^b^ ^***^	3.774 ± 0.170^c^ ^***^
	AA/EDTA/QE 0.5	90.34 ± 0.77^a^ ^***^	99.88 ± 0.36^b^ ^***^	3.934 ± 0.000^c^ ^***^
	AA/EDTA/QE 1	91.35 ± 0.20^a^ ^***^	>100.00^b^ ^***^	3.940 ± 0.000^c^ ^***^
	AA/EDTA/QE 2	91.17 ± 0.57^a^ ^***^	>100.00^b^ ^***^	3.941 ± 0.000^c^ ^***^

-, No activity; S.D., Standard Deviation; ns, Not statistically significant. *p < 0.05, **p < 0.01, ***p < 0.001.

a AA, Ascorbic acid.

b EDTA, Ethylenediamine tetraacetic acid.

c QE, Quercetin.

### Reducing Power Assay

Increasing absorbance values are considered as an indication of an increase in the reducing ability of the samples. The MeOH extract of the plant showed a high absorbance value almost at the same level as the reference compound quercetin at all doses. It was observed that the MeOH extract reached the highest absorbance value, which was determined as 3.930 ± 0.000, at a concentration of 0.5 mg/ml. The absorbance value of quercetin at the same concentration was found to be 3.934 ± 0.000 (Table 1).

### DPPH Radical Scavenging Activity

The DPPH radical scavenging activity of the extractwas over 90.00% at all doses; however, the activity wasn’t dose dependent. Interestingly, the reference compound, ascorbic acid, was also found to have a similar effect profile to MeOH extract. The concentration with the highest DPPH radical scavenging activity was determined as 0.25 mg/ml for both the reference and extract groups, and the inhibition percentages for the ascorbic acid and MeOH extract were calculated as 91.61 ± 0.15 and 91.31 ± 0.58, respectively (Table 1).

### Enzyme Assays

#### *α*-Amylase Inhibitory Activity

*P. atlantica* MeOH extract inhibited the α-amylase enzyme, which has an important role in inhibiting carbohydrate digestion, with a high IC_50_ value of 40.23 ± 3.28 μg/ml, and this value was 1.22 ± 0.17 μg/ml for acarbose. Five sub-extracts (HE, CHCl3, EtOAc, *n*-BuOH and R-H2O) were obtained from the MeOH extract by liquid liquid fractionation. Among these five sub-extracts, EtOAc sub-extract (IC_50=_ 32.78 ± 1.09 μg/ml) showed the highest activity (Table 2). In the light of these data, EtOAc sub-extract was fractionated by polyamide column chromatography, and the obtained seven fractions were evaluated in terms of their effects on the α-amylase enzyme. Among the seven fractions, the glucosidase enzyme inhibitory activity of Fr. G (IC_50_: 33.18 ± 2.88 μg/ml) was found to be quite high (Table 3), and then this fraction was refractionated by RP-18 column chromatography. When the *α*-amylase enzyme inhibitory activities of the RP-18 column chromatography fractions were examined, it was concluded that Fr. G5 (IC_50_: 44.94 ± 10.65) and Fr. G6 (IC_50_: 77.53 ± 31.87) had the highest activity. The IC_50_ value of the reference compound acarbose was determined as IC_50=_ 2.19 ± 0.32 μg/ml (Table 4).

**TABLE 2:** Enzyme inhibitory activity results of *P. atlantica* MeOH and sub-extracts.

Samples	200 μg/ml^a^ inhibition % ± S.D. (IC_50_: µg/ml ± S.D.)			
	α-Amylase	α-Glucosidase	Pancreatic lipase	Pancreatic cholesterol esterase
MeOH extract	98.67 ± 0.56^***^ (IC_50_: 40.22 ± 3.28)	98.94 ± 0.09^***^ (IC_50_: 16.43 ± 1.91)	47.96 ± 4.21^***^ (IC_50_ > 200)	58.98 ± 0.78^***^ (IC_50_: 123.67 ± 0.40)
*n-*HE sub-extract	49.23 ± 1.76^***^ (IC_50_ > 200)	39.18 ± 2.14^***^ (IC_50_ > 200)	0.97 ± 0.65 ^ns^ (IC_50_ > 200)	53.32 ± 0.13^***^ (IC_50_: 61.03 ± 0.11)
CHCl_3_ sub-extract	49.14 ± 2.39^***^ (IC_50_ > 200)	97.16 ± 1.06^***^ (IC_50_: 11.51 ± 4.18)	23.85 ± 3.43^*^ (IC_50_ > 200)	21.55 ± 0.63^***^ (IC_50_ > 200)
EtOAc sub-extract	>100.00^***^ (IC_50_: 32.78 ± 1.09)	95.46 ± 0.48^***^ (IC_50_: 15.44 ± 8.06)	65.81 ± 2.28^***^ (IC_50_: 151.30 ± 4.92)	23.78 ± 1.78^***^ (IC_50_ > 200)
*n*-BuOH sub-extract	83.06 ± 2.33^***^ (IC_50_: 100.18 ± 2.92)	98.20 ± 0.12^***^ (IC_50_: 35.83 ± 1.24)	3.96 ± 3.70 ^ns^ (IC_50_ > 200)	25.68 ± 3.29^***^ (IC_50_ > 200)
R-H_2_O sub-extract	40.89 ± 8.70^***^ (IC_50_ > 200)	37.27 ± 11.07^***^ (IC_50_ > 200)	—	—
References	>100.00^***^ (IC_50_: 1.22 ± 0.17^b^)	99.83 ± 0.32^***^ (IC_50_: 0.025 ± 0.009^b^)	90.61 ± 4.29^***^ (IC_50_: 9.19 ± 4.78^c^)	56.34 ± 1.32^***^ (IC_50_: 142.30 ± 5.67^d^)

-, No activity; S.D., Standard Deviation; ns, Not Statistically Significant. *p < 0.05, **p < 0.01, ***p < 0.001 S.D., Standard Deviation.

a Final concentration.

b Acarbose.

c Orlistat.

d Simvastatin.

**TABLE 3:** Enzyme inhibitory activity results of fractions from polyamide column chromatography of EtOAc sub-extract.

Samples	200 µg/ml^a^ inhibition % ± S.D. (IC_50_: µg/ml ± S.D.)			
	*α*-amylase	*α*-glucosidase	Pancreatic lipase	Pancreatic cholesterol esterase
Fr A	10.42 ± 4.73^ns^ (IC_50_ > 200)	11.75 ± 0.79^***^ (IC_50_ > 200)	-	12.16 ± 2.29^*^ (IC_50_ > 200)
Fr B	56.69 ± 2.62^***^ (IC_50_: 173.55 ± 9.83)	46.16 ± 1.23^***^ (IC_50_ > 200)	-	0.16 ± 3.57^ns^ (IC_50_ > 200)
Fr C	59.09 ± 2.61^***^ (IC_50_: 88.29 ± 209.37	67.85 ± 0.72^***^ (IC_50_: 109.13 ± 0.76)	-	-
Fr D	58.47 ± 3.05^***^ (IC_50_: 187.90 ± 3.82)	34.21 ± 0.35^***^ (IC_50_ > 200)	-	6.87 ± 1.52^ns^ (IC_50_ > 200)
Fr F	90.53 ± 0.91^***^ (IC_50_: 36.38 ± 4.01)	79.87 ± 1.65^***^ (IC_50_: 89.48 ± 2.77)	26.62 ± 2.20^***^ (IC_50_ > 200)	3.03 ± 0.81^ns^ (IC_50_ > 200)
Fr G	85.30 ± 0.44^***^ (IC_50_: 33.18 ± 2.88)	96.90 ± 0.30^***^ (IC_50_: 25.62 ± 0.38)	20.24 ± 4.21^***^ (IC_50_ > 200)	-
References	98.79 ± 0.66^*^ (IC_50_: 1.21 ± 0.18^b^)	99.68 ± 0.15^***^ (IC_50_: 0.05 ± 0.00^b^)	80.94 ± 0.27^***^ (IC_50_: 21.73 ± 2.89^c^)	61.07 ± 0.06^***^ (IC_50_: 79.97 ± 24.84^d^)

-, No activity; S.D., Standard Deviation, ns, Not Statistically Significant *p < 0.05, **p < 0.01, ***p < 0.001. S.D., Standard Deviation.

a Final concentration.

b Acarbose.

c Orlistat.

d Simvastatin.

**TABLE 4:** Enzyme inhibitory activity results of the fractions from RP-18 column chromatography of Fr G.

Samples	*α*-amylase	Samples	*α*-glucosidase
	200 µg/ml^a^ inhibition % ± S.D. (IC_50_: µg/ml ± S.D.)		200 µg/ml^a^ inhibition % ± S.D. (IC_50_: µg/ml ± S.D.)
Fr G1	31.37 ± 2.89^***^ (IC_50_ > 200)	Fr G1	14.73 ± 1.75^***^ (IC_50_ > 200)
Fr G2	35.79 ± 3.65^***^ (IC_50_ > 200)	Fr G2	16.93 ± 0.46^***^ (IC_50_ > 200)
Fr G3	41.93 ± 2.02^***^ (IC_50_ > 200)	Fr G3	—
Fr G4	51.27 ± 1.41^***^ (IC_50_: 144.80 ± 6.40)	Fr G4	55.78 ± 1.61^***^ (IC_50_: 181.00 ± 4.03)
Fr G5	67.23 ± 0.24^***^ (IC_50_: 44.94 ± 10.65	Fr G5	96.80 ± 0.24^***^ (IC_50_: 12.04 ± 0.22)
Fr G6	50.17 ± 1.99^***^ (IC_50_: 77.53 ± 31.87)	Fr G6	93.89 ± 0.83^***^ (IC_50_: 29.66 ± 0.78)
Acarbose	74.51 ± 1.36^***^ (IC_50_: 2.19 ± 0.32)	Acarbose	>100.00^***^ (IC_50_: 0.50 ± 0.01)

-, No activity; S.D., Standard Deviation; *p < 0.05, **p < 0.01, ***p < 0.001.

a Final concentration.

### *α*-Glucosidase Inhibitory Activity

When the glucosidase enzyme inhibitor activities of the MeOH extract and five sub-extracts were tested, it was concluded that the strong inhibitory activity seen in MeOH extract (IC_50=_ 16.14 ± 1.91 μg/ml) was sustained in CHCl_3_ (IC_50=_ 11.51 ± 4.18 μg/ml), EtOAc (IC_50=_ 15.44 ± 8.06 μg/ml), and *n-*BuOH (IC_50=_ 35.83 ± 1.24 μg/ml) sub-extracts after fractionation (Table 2). It was decided to use EtOAc sub-extract for further phytochemical and activity studies since the amount of CHCl_3_ sub-extract with the highest inhibitory activity was low, and its phytochemical profile was similar EtOAc sub-extract by RP-HPLC analysis. The fractions obtained by polyamide column chromatography of the EtOAc sub-extract with the highest activity were evaluated in terms of *α*-glucosidase inhibitory activity. Among the seven fractions, *α-*glucosidase enzyme inhibitory activity of Fr. G (IC_50_: 25.62 ± 0.38 μg/ml) was found to be quite high (Table 3), and then this fraction was refractionated by RP-18 column chromatography. When the *α*-glucosidase enzyme inhibitory activities of the RP-18 column chromatography fractions were examined, it was concluded that Fr. G5 (12.04 ± 0.22) and Fr. G6 (IC_50_: 29.66 ± 0.78) had the highest activity. The IC_50_ value of reference compound acarbose was determined as IC_50=_ 0.50 ± 0.01 μg/ml in this assay (Table 4).

### Pancreatic Lipase Inhibitory Activity

*P. atlantica* MeOH methanol extract and five sub-extracts obtained by the activity-guided fractionation procedure of this extract were tested for *in vitro* pancreatic lipase enzyme inhibitory activity. Except for the EtOAc sub-extract, the IC_50_ values of neither the MeOH extract nor the other sub-extracts could be calculated. The IC_50_ value for orlistat was calculated as 9.20 ± 4.78 μg/ml, while this value was determined as 151.30 ± 4.92 μg/ml for the EtOAc sub-extract (Table 2). When the fractions obtained after the application of EtOAc sub-extract to the polyamide column were evaluated in terms of this activity, it was observed that the effect was completely eliminated in all fractions except Fr. F and Fr. G. (Table 3). Fr. F (26.62 ± 2.20%) and Fr. G (20.24 ± 4.21%) exerted weak pancreatic lipase inhibitory activity at the 2 mg/ml concentration, with orlistat producing 80.94 ± 0.27% inhibition at the same concentration.

### Pancreatic Cholesterol Esterase Inhibitory Activity

It was determined that the pancreatic cholesterol esterase inhibitory activity of the MeOH extract (IC_50=_ 123.67 ± 0.40 μg/ml) was more potent than the reference simvastatin (IC_50=_ 142.33 ± 5.67 μg/ml), and this effect of the HE sub-extract (IC_50=_ 61.03 ± 0.11 μg/ml) obtained by fractionation of the MeOH extract was observed to be much higher (Table 2).

### Protein Tyrosine Phosphatase 1B (PTP1B) Inhibitory Activity Assay

When the MeOH extract and sub-extracts were evaluated in terms of PTP1B inhibitory activity, it was determined that only MeOH extract (9.65 ± 0.94%) and HE sub-extract (14.54 ± 2.26%) showed very weak inhibitory activity at 2 mg/ml concentration. Ursolic acid used as a reference compound produced an inhibition of 59.98 ± 7.19% at 2 mg/ml concentration, while the IC_50_ value was calculated as 1982.00 ± 386.08 μg/ml.

### Determination of Total Phenolic and Flavonoid Content

The total phenolic content of MeOH extract of *P. atlantica* leaves was found 337.9 ± 3.50 mg gallic acid equivalents while the total flavonoid content of methanolic crude leaf extract of *P. atlantica* was was found 16.49 ± 3.00 mg quercetin equivalents per gram. The results indicated that the total phenol content of the MeOH extract was higher than the total flavonoid content.

### Analysis of the MeOH Extract and EtOAc Sub-extract by RP-HPLC

**FIGURE 1:**
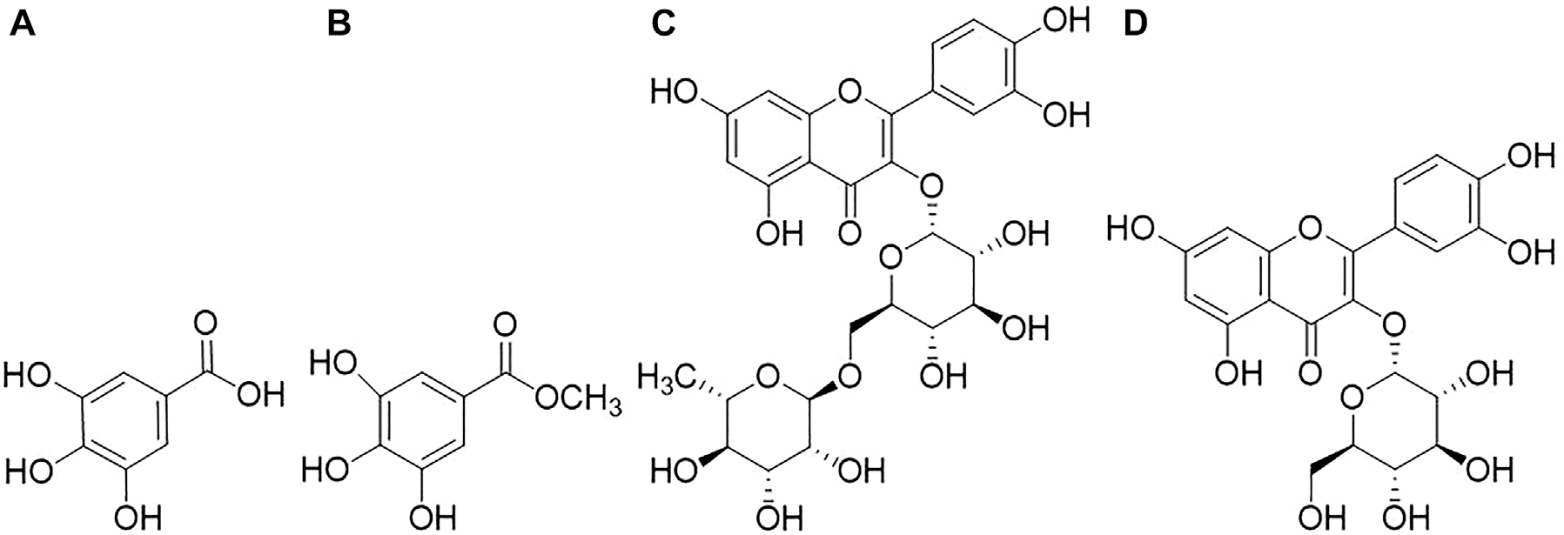
Compounds analyzed qualitatively and quantitatively by RP-HPLC analysis, **(A)**: Gallic acid; **(B)**: Methyl gallate; **(C)**: Rutin; **(D)**: Quercetin-3-*O*-glucoside.

**FIGURE 2:**
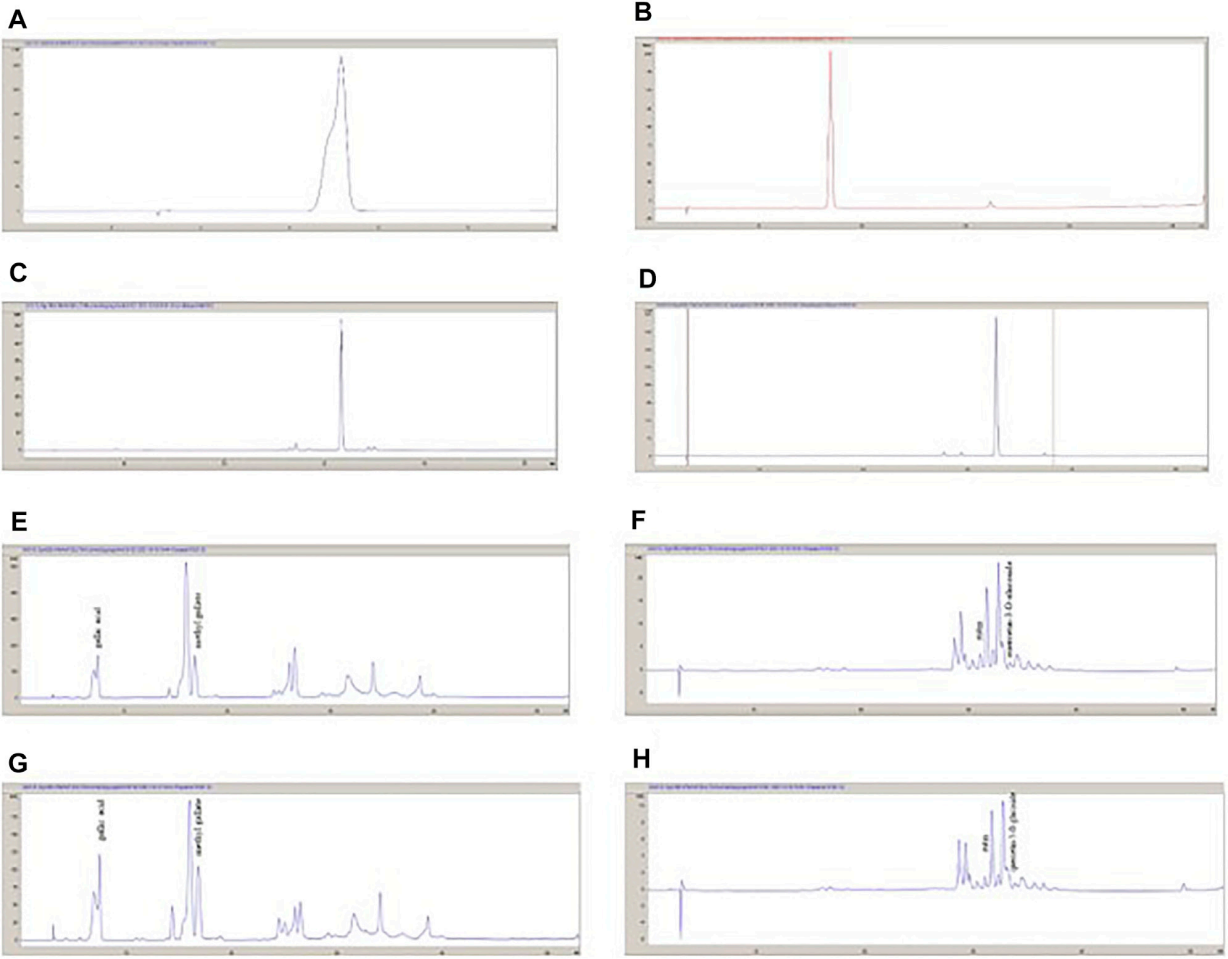
The HPLC chromatograms of standard compounds and the extracts. **(A)**: Gallic acid; **(B)**: Methyl gallate; **(C)**: Rutin; **(D)**: Quercetin-3-*O*-glucoside; **(E)**: *P. atlantica* EtOAc sub-extract (280 nm); **(F)**: *P. atlantica* EtOAc sub-extract (350 nm); **(G)**: *P. atlantica* crude extract (280 nm); **(H)**; *P. atlantica* crude sub-extract (350 nm).

MeOH extract and EtOAc sub-extract were analyzed for phenolic acids and flavonoids by RP-HPLC. Gallic acid, methyl gallate, rutin, and quercetin-3-*O*-glucoside were detected and quantified in both extracts. Methyl gallate, gallic acid, rutin, and quercetin-3-*O*-glucoside (Figure 1) were found in high amounts in EtOAc sub-extract. These compounds first detected in MeOH extract and were intensely extracted with ethyl acetate by fractionation. Methyl gallate was the most abundant phenolic acid present in the EtOAc sub-extract and MeOH extract with the amounts of 8.194 ± 0.016 g/100 g and 5.245 ± 0.014 g/100 g, respectively. Quercetin-3-*O*-glucoside, which was detected in the least amount among the analyzed phenolic compounds, was found as 0.498 ± 0.001 g/100 g in the EtOAc sub-extract and 0.231 ± 0.000 g/100 g in the MeOH extract (Figure 2 and Tables 5, 6).

**TABLE 5:** Retention times (Rt), standard curve and *r* ^2^ values of phenolic compounds.

Compounds	Rt (min)	Standard curve	*r* ^2^
Gallic acid	7.160	y = 75.043x − 10.167	1
Methyl gallate	16.881	y = 40.534x + 55.189	0.9966
Rutin	31.649	y = 57.75x − 69.635	0.9977
Quercetin-3-*O*-glucoside	32.706	y = 68.177x − 6.2647	1

**TABLE 6:** Gallic acid, methyl gallate, rutin, quercetin-3-*O*-glucoside contents of *P. atlantica* MeOH extract and EtOAc sub-extract.

Samples	Compounds (g/100 g dry extract)			
	Gallic acid	Methyl gallate	Rutin	Quercetin-3-*O*-glucoside
EtOAc sub-extract	0.984 ± 0.103	8.194 ± 0.016	0.510 ± 0.001	0.498 ± 0.001
MeOH extract	0.528 ± 0.127	5.245 ± 0.014	0.328 ± 0.000	0.231 ± 0.000

### Characterization of Sub-fractions by LC-QTOF-MS Analysis

**FIGURE 3:**
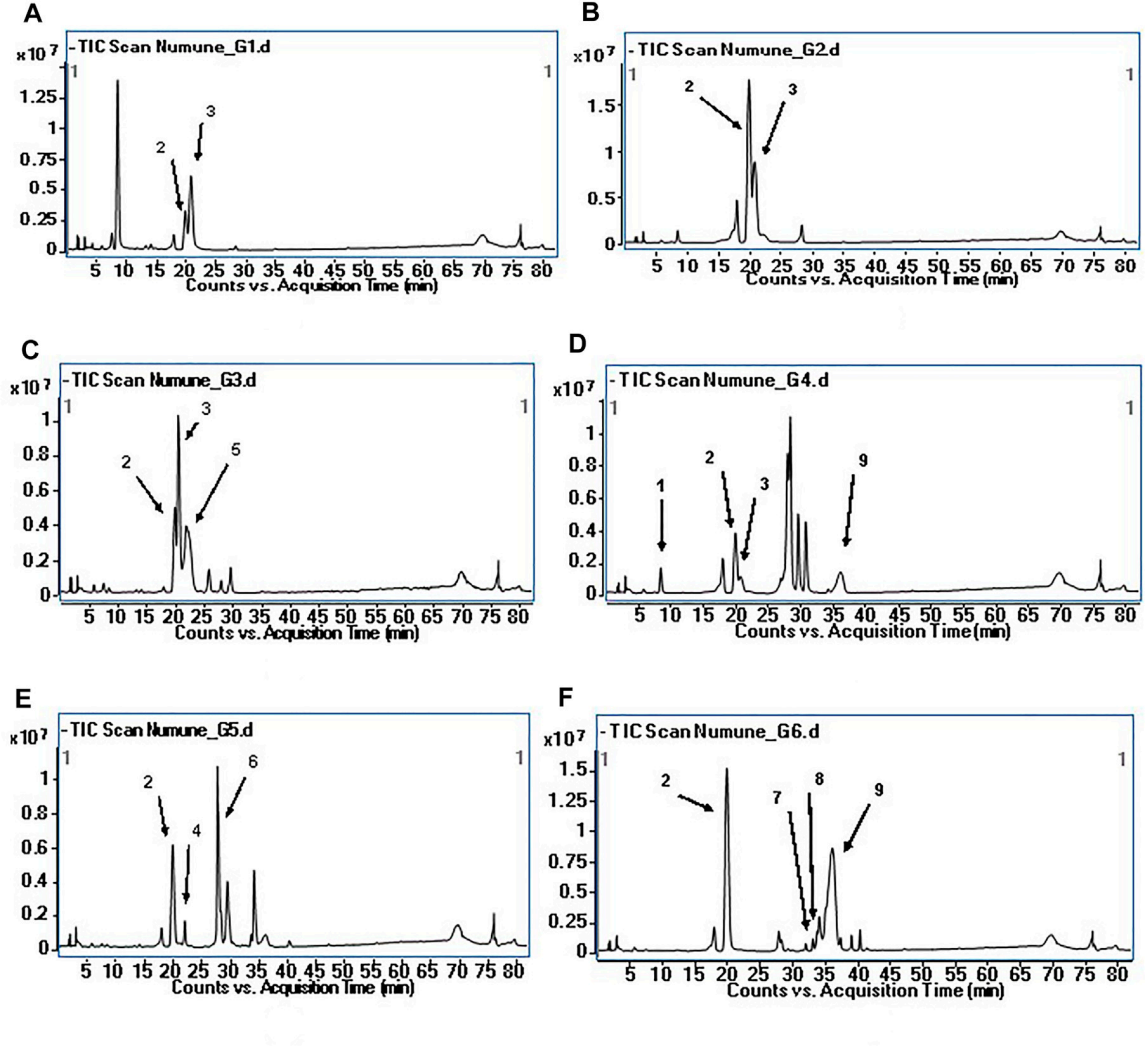
Total ion chromatogram (TIC) of the compounds in G coded sub-fractions **(A)**: TIC of the G1 coded sub-fraction; **(B)**: TIC of the G2 coded sub-fraction; **(C)**: TIC of the G3 coded sub-fraction; **(D)**: TIC of the G4 coded sub-fraction; **(E)**: TIC of the G5 coded sub-fraction; **(F)**: TIC of the G6 coded sub-fraction; The numbers for the molecules indicated on the chromatograms are given in Table 7.

**FIGURE 4:**
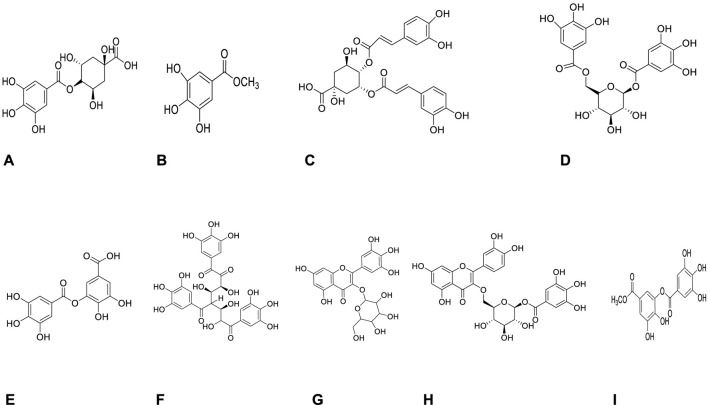
Molecules detected in the G coded sub-fraction by LC-QTOF-MS analysis **(A)**: Galloylquinic acid; **(B)**: Methyl gallate; **(C)**: Digalloylquinic acid; **(D)**: Digalloylglucose; **(E)**: Digallic acid; **(F)**: Trigalloylglucose; **(G)**: Myricetin-3-O-hexoside; **(H)**: 2″-O-galloyl-quercetin-3-O-hexoside; **(I)**: Methyl digallate.

**FIGURE 5:**
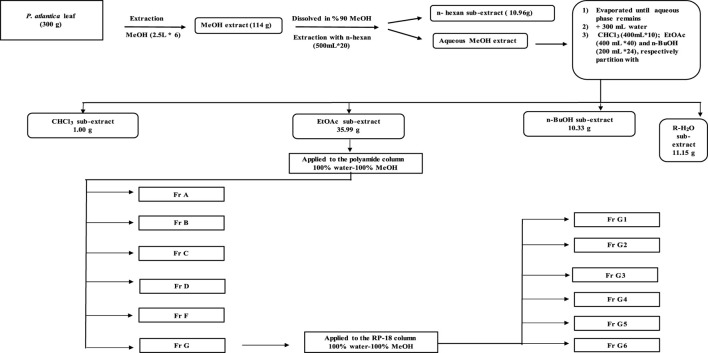
Activity-guided fractionation flow chart.

Phytochemical characterization of fraction-G obtained from RP-18 column chromatography was performed by LC-QTOF-MS analysis. Figure 3 shows the phytochemical content of six G-coded sub-fractions. A total of nine phenolic compounds were identified and characterized. Methyl gallate was detected in all G sub-fractions. Digalloylquinic acid in sub-fractions G1, G2, G3, G4; galloylquinic acid and methyl digallate in sub-fraction G4; digallic acid in sub-fraction G3; digalloylglucose and trigalloylglucose glucose in sub-fraction G5, and myricetin-3-*O*-hexoside, 2″-*O*-galloyl-quercetin-3-*O*-hexoside, and methyl digallate in sub-fraction G6 were characterized (Figures 4, 5). In addition to these, thirteen unknown compounds were determined in the G coded sub-fractions (Table 7).

**TABLE 7:** LC-QTOF-MS characterisation of compounds in G coded sub-fractions.

Detectable compounds								
Compound number	Retention time (min)	Molecular formula	Molecular weight	[M-H]^−^	Fragment ions	Mass error (ppm)	Compound	Sub-fractions
1	8.40	C_14_H_16_O_10_	344.07435	343.0680	191	2.49	Galloylquinic acid	G4
2	19.74	C_8_H_8_O_5_	184.03717	183.0299	124, 78	0.26	Methyl gallate	G1, G2, G3, G4, G5, G6
3	20.75	C_21_H_20_O_14_	496.08531	495.0785	343, 191	0.60	Digalloylquinic acid	G1, G2, G3, G4
4	21.94	C_20_H_20_O_14_	484.08531	483.0787	271, 169	0.99	Digalloylglucose	G5
5	22.24	C_14_H_10_O_9_	322.03248	321.0258	169	1.46	Digallic acid	G3
6	27.81	C_27_H_24_O_18_	636.09626	635.0895	509, 169	0.88	Trigalloylglucose	G5
7	31.98	C_21_H_20_O_13_	480.09039	479.0835	315	0.46	Myricetin-3-*O*-hexoside	G6
8	33.08	C_28_H_24_O_16_	616.10644	615.1001	463, 301	1.26	2″-*O*-galloyl-quercetin-3-*O-*hexoside	G6
9	36.10	C_15_H_12_O_9_	336.04810	335.0410	183, 124	0.24	Methyl digallate	G4, G6
Undetectable compounds								
	8.50			687.2500	343, 191		Unknown compound	G1
	17.73			357.0495	169, 125		Unknown compound	G2, G4, G6
	22.25			320.9981	169		Unknown compound	G2
	25.81			477.0102	325, 169		Unknown compound	G3
	28.29			509.0288	357, 169		Unknown compound	G2, G3, G4, G5, G6
	29.571			646.9949	495, 343, 169		Unknown compound	G3, G4
	34.09			661.0104	509, 330, 254		Unknown compound	G6
	34.29			661.0086	509, 169		Unknown compound	G5
	34.991			441.0357	335, 289, 183		Unknown compound	G6
	37.35			713.3686	677, 599		Unknown compound	G6
	38.96			826.4267	790		Unknown compound	G6
	40.26			477.0096	301		Unknown compound	G5
	40.31			963.5256	939, 903		Unknown compound	G6

A total of nine compounds were detected in the G-coded sub-fraction. These are galloylquinic acid with *m/z* 343.0680 [M-H]^−^ and its fragment ion *m/z* 191; methyl gallate *m/z* 183.0299 [M-H]^−^ with fragment ions *m/z* 124 and 78, digalloylquinic acid *m/z* 495.0785 [M-H]^−^ with fragment ions *m/z* 343 and 191; digalloylglucose *m/z* 483.0787 [M-H]^−^ with fragment ions *m/z* 271 and 169; digallic acid *m/z* 321.0258 [M-H]^−^ with fragment ion *m/z* 169; trigalloylglucose *m/z* 635.0895 [M-H]^−^ with fragment ions *m/z* 509 and 169; myricetin-3-*O*-hexoside *m/z* 479.0835 [M-H]^−^ with fragment ion *m/z* 315; 2″-*O*-galloyl-quercetin-3-*O*-hexoside *m/z* 615.1001 [M-H]^−^ with fragment ions *m/z* 463 and 301; finally methyl digallate *m/z* 335.0410 [M-H]^−^ fragment ions *m/z* 183 and 124 (Figure 3 and Table 7). In addition, when the fragment ions of the compounds thought to have this molecular weight and the fragment ions belonging to the peaks thought to belong to these compounds in sub-fractions were compared, it was predicted that these might be the compounds mentioned above.

## Discussion

This study tested the potential antidiabetic and antiobesity effects of the methanol extract of *P. atlantica* leaves, the sub-extract fraction, and sub-fractions obtained from this extract by activity-guided fractionation by *in vitro* enzyme inhibition methods. In the light of the findings gained by the activity studies, the phytochemical contents of the most active fractions were analyzed with RP-HPLC and LC-QTOF-MS methods. *P. atlantica* methanolic leaf extract showed strong effects on *α*-glucosidase, *α*-amylase, and pancreatic cholesterol esterase enzymes. Since diabetes mellitus is a metabolic disorder that causes oxidative stress in the organism, the antioxidant potential of MeOH extract was evaluated. A strong DPPH radical scavenging effect and ferric reducing capacity suggested that the methanolic extract may effectively combat oxidative stress in diabetes mellitus. Among the fractions obtained by activity-guided fractionation, the IC_50_ value of EtOAc sub-extract on α-glucosidase, α-amylase, and pancreatic lipase enzymes was calculated to be lower than that of the MeOH extract. The potent pancreatic cholesterol esterase enzyme inhibitory effect of the MeOH extract was detected with a similar inhibition value in the HE sub-extract, indicating that the compounds responsible for the effect may be due to non-polar compounds. It was reported for the first time that *P. atlantica* leaf methanol extract and its sub-extracts did not have a significant effect against the PTP1B enzyme, which has an essential role in diabetes and obesity and is a negative regulator of leptin and insulin signaling pathways. It was determined by RP-HPLC that MeOH extract and the most active EtOAc sub-extract were rich in methyl gallate and containing gallic acid, rutin, and quercetin-3-*O*-glucoside.

EtOAc sub-extract, which was determined to have the strongest effect against the three enzymes (*α*-glucosidase, *α*-amylase, and pancreatic lipase) among the sub-extracts, was fractionated by open column chromatography using different column adsorbents (polyamide and RP18). As a result of chromatographic fractionation procedures, it was concluded that the pancreatic lipase inhibitory activity of EtOAc sub-extract was highly reduced and this effect was due to synergism. On the other hand, it was observed that the inhibitory effects of the obtained sub-fractions on *α*-glucosidase and *α*-amylase became much more pronounced. Among the fractions obtained by polyamide column chromatography, the G-coded fraction potently inhibited both *α-*amylase and α-glucosidase enzymes. Subsequently, this fraction was refractionated by RP-18 column chromatography, and it was found that the effect on both enzymes increased significantly in the G5 coded fraction. In the light of all these results LC-QTOF-MS was used to determine the phytochemical content of this sub-fraction and other G-coded fractions. In these sub-fractions nine known and thirteen unknown compounds were detected.

To date, numerous studies of antioxidant activity have been conducted on *P. atlantica* leaves. Antioxidant activities of aqueous, 80% methanolic, 70% acetone, ethyl acetate, and ethanolic extracts prepared from samples collected from Algeria by different researchers were evaluated using different methods. In these studies, mostly the leaves showed high antioxidant activity. Although it is generally thought that the flavonoids and phenols contained in the leaves are responsible for the antioxidant activity of the plant, studies have been carried out to determine the compounds responsible for the effect with further instrumental analysis techniques (HPLC and LC-MS) (Benhammou et al., 2008). As a result of these analyzes, the presence of phenolic compounds such as gallic acid, digallic, cichoric, quinic, gentisic, vanillic, protocatechuic, rosmarinic, galloylquinic, digalloylquinic, trigalloylquinic, tetragalloylquinic, galloylshikimic acids, trigalloylglucose, tetragalloylglucose, rutin catechin, quercetin, methyl gallate, and glucogallin in the leaves of the plant was determined (Benhammou et al., 2008; Ahmed et al., 2016; Toul et al., 2017; Benamar et al., 2018; Achili et al., 2020). On the other hand, Peksel et al. (2010) evaluated the antioxidant potential of methanol and ethyl acetate extracts of *P. atlantica* leaves collected from Istanbul. The reducing power, metal chelating activity, DPPH radical scavenging activity, and total antioxidant capacity were evaluated in these extracts. While the antioxidant activities of the methanolic extract were higher than the ethyl acetate extract at the same concentrations, it was stated that the ethyl acetate extract had higher metal chelating activity. It has been reported that both extracts have strong free radical scavenging activity, and their antioxidant capacity may be related to their chemical composition (Peksel et al., 2010). Rigane et al. (2017) investigated the antioxidant potential of aqueous and ethanolic extracts of *P. atlantica* leaves collected from Tunisia. It is stated that the total amount of phenolic compounds in both extracts is higher than the total amount of flavonoids. The DPPH radical scavenging activity of the extracts has been reported to have an IC_50_ concentration of 32–200 μg/ml. The study also reported a good correlation between total phenolic content and radical scavenging activities (Rigane et al., 2017). In our study, apart from the metal chelating activity of methanol extract of *P. atlantica* leaves, both DPHH radical scavenging and ferric reducing power activities were found to be close to those of the reference compounds.

As a result of the literature survey, it has been determined that there are *in vitro* and *in vivo* antidiabetic activity studies with aqueous and 70% acetone extracts of *P. atlantica* leaves. In studies where *in vitro α*-amylase and *α*-glucosidase enzyme inhibitory effects of the plant leaves were evaluated, only aqueous or 70% acetone extracts of the leaves were tested (Hamdan and Afifi, 2004). investigated the *in vitro α*-amylase inhibitory activities of aqueous extracts of *P. atlantica, Ferula persica, Paronychia argentea*. In addition, the hypoglycemic activities of these extracts were tested in streptozocin-induced hyperglycemic rats. These plants, which are used in the traditional herbal medicine of Jordan for diabetes, did not show any significant hypoglycemic activity in diabetic animals.

In conclusion, it has been reported that *P. argentea* and *P. atlantica* show potent *α*-amylase inhibitory activity *in vitro* (Hamdan and Afifi, 2004). Kasabri et al. (2011) evaluated the antidiabetic potential of aqueous extracts of *P. atlantica, Achillea santolina* L., *Rheum ribes* L., *Sarcopoterium spinosum* (L.) Spach, and *Teucrium polium* L. by *in vitro* and *in vivo* methods. Compared with acarbose, *P. atlantica* aqueous extract significantly inhibited *α*-amylase and *α*-glucosidase in a dose-dependent manner, while improving glucose intolerance in glucose-loaded fasted rats. In the light of the results, *P. atlantica* was suggested as a potential candidate for the amelioration/management of type 2 diabetes (Kasabri et al., 2011). In these two studies, no comments were made on the compounds responsible for the enzyme inhibitory activities of *P. atlantica* leaf extracts.

(Ahmet et al., 2018) investigated *α*-amylase and *α*-glucosidase enzyme inhibitory activities of the ethyl acetate sub-extract obtained from 70% acetone extract of *P. atlantica* leaves. By comparing the model’s regression coefficients with the fingerprints' peaks, the peaks potentially responsible for the inhibition of *α*-amylase and *α*-glucosidase enzymes were tried to be determined. For this purpose, Partial Least Squares regression and fingerprints Correlation Optimized Warping methods were used. According to the analysis data, the researchers suggested that glucogallin, quinic acid, and galloylquinic acid inhibit only *α*-amylase; methyl gallate and tetragalloylglucose inhibit only *α*-glucosidase, while, gallic acid, gentisic acid, and digalloylquinic acid inhibit both *α*-amylase and *α*-glucosidase. In our study, sub-extracts and fractions were obtained from *P. atlantica* leaf methanol extract by activity-guided fractionation and various chromatographic methods, and their enzyme activities were evaluated at each step. Trigalloylglucose, digalloylglucose, methyl gallate, and three unknown compounds were determined by LC-QTOF-MS in the G5 coded sub-fraction, which strongly inhibited both enzymes. Nevertheless, quinic acid, gentisic acid, glucogallin and tetragalloylglucose could not be detected in any of the G-coded sub-fractions (Li et al., 2019). reported that 3,4,6-tri-*O*-galloyl-*D*-glucose dose-dependently inhibited *α*-amylase and *α*-glucosidase enzymes with IC_50_ values of 334.6 ± 8.9 and 46.5 ± 1.6 μM, respectively (Li et al., 2019). There are scientific studies on the inhibitory potentials of methyl gallate on *α*-amylase and *α*-glucosidase enzymes (Wansi et al., 2007; Li et al., 2009; Dubey et al., 2017; El Souda et al., 2017; Bamigboye et al., 2020). On the other hand, there is no published scientific article about the inhibitory effects of methyl digallate, 2″-*O*-galloyl-quercetin-3-*O*-hexoside, myricetin-3-*O*-hexoside, and digalloylglucose on these two enzymes. In addition to the G5 coded sub-fraction, a strong *α-*glucosidase enzyme inhibitory activity was also observed in the G6 coded sub-fraction. In this sub-fraction, methyl gallate, methyl digallate, 2″-*O*-galloyl-quercetin-3-*O*-hexoside, myricetin-3-*O*-hexoside, and seven unknown compounds were identified. Interestingly, as shown in Figure 3, the G-coded sub-fractions containing digalloylquinic acid were determined to have very weak or no *α-*glucosidase and *α-*amylase inhibitory effects. This finding suggested that digalloylquinic acid may cause antagonistic effects. Many of the compounds (methyl gallate, methyl digallate, 2″-*O*-galloyl-quercetin-3-*O*-hexoside, myricetin-3-*O*-hexoside, trigalloylglucose, digalloylglucose) that we predicted to be responsible for the *α*-glucosidase inhibitory activity of *P. atlantica* leaf extract was interpreted for the first time in this study in terms of this effect.

## Conclusion

To summarize, the strong *α*-glucosidase, *α*-amylase inhibitor and antioxidant effects of the methanol extract of *P. atlantica* leaves showed that the extract might provide significant benefits against oxidative stress caused by diabetes in tissues and organs. The isolation of phenolic compounds, which are thought to be responsible for the effect in the sub-fractions obtained by activity-guided fractionation studies, is important and necessary. However, it should be also considered that these compounds may cause a strong antidiabetic effect with a synergistic effect. For this reason, the methanol extract of the plant can be standardized by RP-HPLC on the basis of methyl gallate as a marker compound and presented to human health as a traditional herbal medicinal product for the treatment of diabetes mellitus. On the other hand, the effects of *P. atlantica* leaves on pancreatic lipase, pancreatic cholesterol esterase, and PTP1B enzymes were evaluated for the first time. The methanol extract and *n*-hexane sub-extract of the leaves showed much stronger effects on the pancreatic cholesterol esterase enzyme than simvastatin. Since it will be very important that the antidiabetic compounds have strong antioxidant and antihyperlipidemic effects, further studies are needed to evaluate the effects of the plant on the cholesterol esterase enzyme and isolate the effective compounds.

## Data Availability

The original contributions presented in the study are included in the article/Supplementary Material, further inquiries can be directed to the corresponding author.
